# [5,5′-Dihydr­oxy-2,2′-[*o*-phenyl­enebis(nitrilo­methyl­idyne)]diphenolato}copper(II) methanol disolvate

**DOI:** 10.1107/S1600536809053720

**Published:** 2009-12-19

**Authors:** Meiju Niu, Shumei Fan, Kai Liu, Zhiqiang Cao, Daqi Wang

**Affiliations:** a College of Chemistry and Chemical Engineering, Liaocheng University, Shandong 252059, People’s Republic of China; b Dongchang College Liaocheng University, Shandong 252000, People’s Republic of China

## Abstract

In the title compound, [Cu(C_20_H_14_N_2_O_4_)]·2CH_3_OH, the Cu^II^ ion is coordinated by two N [Cu—N = 1.933 (2) and 1.941 (2) Å] and two O [Cu—O = 1.890 (2) and 1.9038 (19) Å] atoms from the tetra­dentate Schiff base ligand 5,5′-dihydr­oxy-2,2′-[*o*-phenyl­enebis(nitrilo­methyl­idyne)]diphen­olate (*L*) in a distorted square-planar geometry. In the crystal, inter­molecular O—H⋯O hydrogen bonds link two Cu*L* mol­ecules and four solvent mol­ecules into a centrosymmetric cluster. The crystal packing exhibits short inter­molecular C⋯C contacts of 3.185 (4) and 3.232 (4) Å.

## Related literature

For related structures, see: Amirnasr *et al.* (2006 ▶); Arola-Arnal *et al.* (2008 ▶); Sundaravel *et al.* (2009 ▶); Lu *et al.* (2006 ▶).
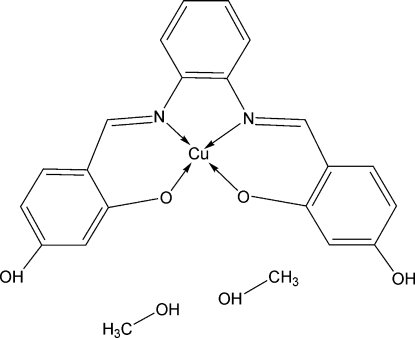


## Experimental

### 

#### Crystal data

[Cu(C_20_H_14_N_2_O_4_)]·2CH_4_O
*M* *_r_* = 473.96
Triclinic, 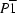

*a* = 7.9520 (17) Å
*b* = 11.066 (2) Å
*c* = 11.870 (2) Å
α = 91.796 (2)°
β = 94.604 (3)°
γ = 94.241 (3)°
*V* = 1037.6 (4) Å^3^
*Z* = 2
Mo *K*α radiation
μ = 1.10 mm^−1^
*T* = 293 K
0.53 × 0.48 × 0.21 mm

#### Data collection

Bruker SMART 1000 CCD area-detector diffractometer
Absorption correction: multi-scan (*SADABS*; Sheldrick, 1996 ▶) *T*_min_ = 0.595, *T*_max_ = 0.803
5360 measured reflections
3592 independent reflections
2873 reflections with *I* > 2σ(*I*)
*R*_int_ = 0.027

#### Refinement

*R*[*F* ^2^ > 2σ(*F* ^2^)] = 0.037
*wR*(*F* ^2^) = 0.099
*S* = 1.00
3592 reflections
280 parameters
H-atom parameters constrained
Δρ_max_ = 0.46 e Å^−3^
Δρ_min_ = −0.36 e Å^−3^

### 

Data collection: *SMART* (Siemens, 1996 ▶); cell refinement: *SAINT* (Siemens, 1996 ▶); data reduction: *SAINT*; program(s) used to solve structure: *SHELXS97* (Sheldrick, 2008 ▶); program(s) used to refine structure: *SHELXL97* (Sheldrick, 2008 ▶); molecular graphics: *SHELXTL* (Sheldrick, 2008 ▶); software used to prepare material for publication: *SHELXTL*.

## Supplementary Material

Crystal structure: contains datablocks I, global. DOI: 10.1107/S1600536809053720/cv2670sup1.cif

Structure factors: contains datablocks I. DOI: 10.1107/S1600536809053720/cv2670Isup2.hkl

Additional supplementary materials:  crystallographic information; 3D view; checkCIF report

## Figures and Tables

**Table 1:** Hydrogen-bond geometry (Å, °)

*D*—H⋯*A*	*D*—H	H⋯*A*	*D*⋯*A*	*D*—H⋯*A*
O2—H2⋯O5^i^	0.82	1.91	2.704 (3)	163
O4—H4⋯O6^ii^	0.82	1.82	2.602 (4)	159
O5—H5⋯O4^iii^	0.82	1.97	2.788 (3)	176
O6—H6⋯O1	0.82	2.18	2.777 (3)	130
O6—H6⋯O3	0.82	2.34	3.037 (4)	144

Symmetry codes: (i) 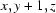; (ii) 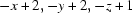; (iii) 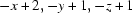.

**Fig. 1.**
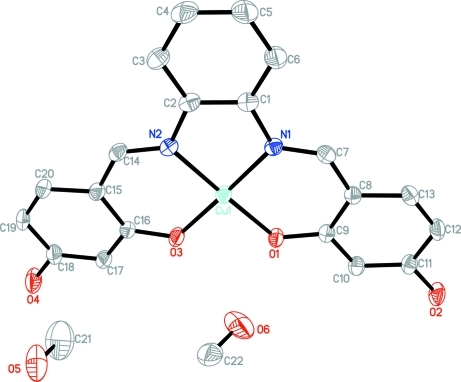
The molecular structure of the title compound showing the atomic labels and 30% probability displacement ellipsoids.

## supplementary crystallographic information

### Comment

Copper complexes have attracted intensive interest in the past decade because they play important roles on the fields of coordination chemistry, bioinorganic chemistry, redox enzyme systems and others (Amirnasr *et al.*, 2006). In a continuation of a study of Schiff base ligands and their copper(II) complexes, we report here the title complex, (I).

In (I) (Fig. 1),the main plane being formed by the three phenyl and the N_2_O_2_. The angles O1—Cu1—N2 (178.64 (9)°) and O3—Cu1—N1 (178.39 (9)°) indicate that the coordination geometry of the copper atom is four-coordinate in an approximately square planar, which acts as a tetradentate ligand through its *o*-phenylenediamine N atoms and its deprotonated phenol O atoms. This square planar geometry is the most usual for Cu^II^ complexes (Arola-Arnal *et al.*, 2008) in the N_2_0_2_ donor set with Schiff base ligands. The Cu—O distances of 1.9022 (19)Å and 1.889 (2)Å are very close to the corresponding values in related structures (1.904 (2)Å and 1.884 (3) Å; Sundaravel *et al.*, 2009). The Cu—N distances of 1.932 (2)Å and 1.942 (2)Å are very close to the corresponding values in related structures(1.946 (2) Å; Lu *et al.*, 2006). Intermolecular O—H···O hydrogen bonds (Table 1) link the molecules into a centrosymmetric cluster.

### Experimental

*o*-Phenylenediamine(1 mmol, 108.22 mg) was dissolved in hot ethanol (20 ml) and added dropwise to a ethanol solution (10 ml) of 2,4-dihydroxybenzaldehyde (2 mmol, 276.2 mg). The mixture was then stirred at 323 K for 4 h. The triethylamine solution (3 ml) of Copper (II) acetate (1.5 mmol, 299.5 mg) was then added dropwise and the mixture stirred for another 5 h, at which point a red precipitate collected by suction filtration and washed with ethanol and ether. Crystals of the title compound suitable for X-ray analysis were from the methanol and dimethylformamide solution after about two weeks.

### Refinement

All H atoms were placed geometrically and treated as riding on their parent atoms with C—H = 0.96 Å (methylene) or 0.93 Å (aromatic), 0.82 Å (hydroxyl) and *U*_iso_(H) = 1.2-1.5*U*_eq_ of the parent atom.

### Crystal data

**Table:**

[Cu(C_20_H_14_N_2_O_4_)]·2CH_4_O	*Z* = 2
*M**_r_* = 473.96	*F*(000) = 490
Triclinic, *P*1	*D*_x_ = 1.517 Mg m^−^^3^
*a* = 7.9520 (17) Å	Mo *K*α radiation, λ = 0.71073 Å
*b* = 11.066 (2) Å	Cell parameters from 2490 reflections
*c* = 11.870 (2) Å	θ = 2.5–26.4°
α = 91.796 (2)°	µ = 1.10 mm^−^^1^
β = 94.604 (3)°	*T* = 293 K
γ = 94.241 (3)°	Block, red
*V* = 1037.6 (4) Å^3^	0.53 × 0.48 × 0.21 mm

### Data collection

**Table:**

Bruker SMART 1000 CCD area-detector diffractometer	3592 independent reflections
Radiation source: fine-focus sealed tube	2873 reflections with *I* > 2σ(*I*)
graphite	*R*_int_ = 0.027
phi and ω scans	θ_max_ = 25.0°, θ_min_ = 1.7°
Absorption correction: multi-scan (*SADABS*; Sheldrick, 1996)	*h* = −9→9
*T*_min_ = 0.595, *T*_max_ = 0.803	*k* = −12→13
5360 measured reflections	*l* = −14→10

### Refinement

**Table:**

Refinement on *F*^2^	Primary atom site location: structure-invariant direct methods
Least-squares matrix: full	Secondary atom site location: difference Fourier map
*R*[*F*^2^ > 2σ(*F*^2^)] = 0.037	Hydrogen site location: inferred from neighbouring sites
*wR*(*F*^2^) = 0.099	H-atom parameters constrained
*S* = 1.00	*w* = 1/[σ^2^(*F*_o_^2^) + (0.0446*P*)^2^ + 0.5623*P*] where *P* = (*F*_o_^2^ + 2*F*_c_^2^)/3
3592 reflections	(Δ/σ)_max_ < 0.001
280 parameters	Δρ_max_ = 0.46 e Å^−^^3^
0 restraints	Δρ_min_ = −0.36 e Å^−^^3^

### Fractional atomic coordinates and isotropic or equivalent isotropic displacement parameters (Å^2^)

**Table:**

	*x*	*y*	*z*	*U*_iso_*/*U*_eq_	
Cu1	0.84950 (4)	0.91865 (3)	0.07516 (3)	0.03348 (14)	
N1	0.7711 (3)	0.9467 (2)	−0.07955 (19)	0.0322 (5)	
N2	0.9482 (3)	0.7807 (2)	0.00880 (19)	0.0317 (5)	
O1	0.7530 (2)	1.05584 (16)	0.13697 (16)	0.0356 (5)	
O2	0.4653 (3)	1.41526 (18)	0.14493 (19)	0.0522 (6)	
H2	0.4991	1.4142	0.2120	0.078*	
O3	0.9320 (3)	0.89012 (18)	0.22501 (16)	0.0433 (5)	
O4	1.2805 (4)	0.7377 (2)	0.52128 (19)	0.0724 (8)	
H4	1.2474	0.7960	0.5551	0.109*	
O5	0.6076 (4)	0.4576 (2)	0.3584 (2)	0.0780 (8)	
H5	0.6454	0.4010	0.3927	0.117*	
O6	0.7454 (6)	1.0573 (3)	0.3705 (3)	0.1286 (17)	
H6	0.8050	1.0433	0.3191	0.193*	
C1	0.8192 (4)	0.8588 (3)	−0.1582 (2)	0.0374 (7)	
C2	0.9139 (4)	0.7681 (3)	−0.1106 (3)	0.0373 (7)	
C3	0.9676 (4)	0.6789 (3)	−0.1807 (3)	0.0455 (8)	
H3	1.0314	0.6191	−0.1498	0.055*	
C4	0.9275 (5)	0.6780 (3)	−0.2961 (3)	0.0581 (9)	
H4A	0.9624	0.6170	−0.3425	0.070*	
C5	0.8358 (6)	0.7674 (3)	−0.3423 (3)	0.0670 (11)	
H5A	0.8101	0.7672	−0.4201	0.080*	
C6	0.7815 (5)	0.8574 (3)	−0.2742 (3)	0.0561 (9)	
H6A	0.7193	0.9174	−0.3063	0.067*	
C7	0.6728 (4)	1.0312 (3)	−0.1095 (2)	0.0351 (7)	
H7	0.6340	1.0315	−0.1855	0.042*	
C8	0.6194 (3)	1.1225 (2)	−0.0376 (2)	0.0318 (6)	
C9	0.6652 (3)	1.1344 (2)	0.0815 (2)	0.0314 (6)	
C10	0.6127 (4)	1.2337 (2)	0.1415 (3)	0.0366 (7)	
H10	0.6430	1.2431	0.2188	0.044*	
C11	0.5170 (4)	1.3183 (3)	0.0890 (3)	0.0378 (7)	
C12	0.4682 (4)	1.3056 (3)	−0.0271 (3)	0.0436 (8)	
H12	0.4022	1.3618	−0.0624	0.052*	
C13	0.5181 (4)	1.2108 (3)	−0.0873 (3)	0.0395 (7)	
H13	0.4849	1.2029	−0.1643	0.047*	
C14	1.0398 (4)	0.7072 (2)	0.0662 (2)	0.0357 (7)	
H14	1.0792	0.6432	0.0259	0.043*	
C15	1.0849 (4)	0.7158 (2)	0.1845 (2)	0.0361 (7)	
C16	1.0355 (4)	0.8091 (2)	0.2577 (2)	0.0356 (7)	
C17	1.1031 (4)	0.8149 (3)	0.3713 (3)	0.0444 (8)	
H17	1.0741	0.8761	0.4198	0.053*	
C18	1.2108 (4)	0.7320 (3)	0.4118 (3)	0.0487 (8)	
C19	1.2564 (4)	0.6381 (3)	0.3410 (3)	0.0500 (8)	
H19	1.3279	0.5813	0.3690	0.060*	
C20	1.1945 (4)	0.6318 (3)	0.2312 (3)	0.0438 (8)	
H20	1.2253	0.5696	0.1844	0.053*	
C21	0.7229 (7)	0.5589 (4)	0.3737 (5)	0.1056 (17)	
H21A	0.7013	0.6045	0.4404	0.158*	
H21B	0.7110	0.6087	0.3092	0.158*	
H21C	0.8358	0.5332	0.3820	0.158*	
C22	0.6689 (6)	0.9506 (5)	0.4033 (4)	0.0857 (14)	
H22A	0.6713	0.9513	0.4843	0.129*	
H22B	0.7282	0.8840	0.3772	0.129*	
H22C	0.5537	0.9418	0.3714	0.129*	

### Atomic displacement parameters (Å^2^)

**Table:**

	*U*^11^	*U*^22^	*U*^33^	*U*^12^	*U*^13^	*U*^23^
Cu1	0.0365 (2)	0.0294 (2)	0.0349 (2)	0.00663 (14)	0.00252 (14)	−0.00080 (14)
N1	0.0339 (13)	0.0275 (12)	0.0353 (13)	0.0012 (10)	0.0054 (10)	−0.0027 (10)
N2	0.0280 (12)	0.0304 (12)	0.0360 (13)	−0.0005 (10)	0.0034 (10)	−0.0040 (10)
O1	0.0411 (11)	0.0303 (10)	0.0356 (11)	0.0106 (9)	−0.0022 (9)	0.0002 (8)
O2	0.0675 (15)	0.0326 (12)	0.0578 (14)	0.0219 (11)	−0.0016 (11)	−0.0014 (10)
O3	0.0554 (14)	0.0415 (12)	0.0356 (11)	0.0245 (10)	0.0028 (10)	−0.0013 (9)
O4	0.111 (2)	0.0646 (17)	0.0450 (14)	0.0490 (16)	−0.0096 (14)	0.0024 (12)
O5	0.113 (2)	0.0505 (16)	0.0681 (17)	0.0266 (16)	−0.0249 (16)	−0.0026 (13)
O6	0.272 (5)	0.072 (2)	0.0547 (19)	0.053 (3)	0.061 (3)	−0.0016 (16)
C1	0.0372 (16)	0.0361 (16)	0.0380 (17)	−0.0040 (13)	0.0053 (13)	−0.0033 (13)
C2	0.0354 (16)	0.0331 (15)	0.0423 (17)	−0.0042 (13)	0.0070 (13)	−0.0082 (13)
C3	0.0447 (19)	0.0407 (18)	0.050 (2)	0.0028 (14)	0.0047 (15)	−0.0109 (15)
C4	0.073 (3)	0.051 (2)	0.051 (2)	0.0060 (18)	0.0110 (18)	−0.0194 (17)
C5	0.101 (3)	0.065 (2)	0.0360 (19)	0.018 (2)	0.0022 (19)	−0.0094 (17)
C6	0.080 (3)	0.050 (2)	0.0397 (19)	0.0174 (18)	0.0036 (17)	−0.0033 (16)
C7	0.0366 (16)	0.0346 (16)	0.0330 (16)	−0.0040 (13)	0.0020 (13)	0.0027 (13)
C8	0.0290 (15)	0.0286 (14)	0.0373 (16)	−0.0023 (12)	0.0026 (12)	0.0047 (12)
C9	0.0265 (14)	0.0266 (14)	0.0402 (16)	−0.0030 (11)	0.0006 (12)	0.0020 (12)
C10	0.0374 (17)	0.0301 (15)	0.0416 (17)	0.0024 (12)	−0.0008 (13)	0.0009 (13)
C11	0.0348 (16)	0.0283 (15)	0.0498 (18)	0.0011 (12)	0.0006 (14)	0.0046 (13)
C12	0.0433 (18)	0.0337 (16)	0.055 (2)	0.0091 (14)	0.0009 (15)	0.0154 (15)
C13	0.0403 (17)	0.0387 (17)	0.0388 (16)	−0.0007 (14)	−0.0011 (13)	0.0100 (14)
C14	0.0351 (16)	0.0257 (14)	0.0468 (18)	0.0017 (12)	0.0097 (13)	−0.0053 (13)
C15	0.0395 (17)	0.0271 (15)	0.0429 (17)	0.0043 (12)	0.0090 (13)	0.0015 (13)
C16	0.0384 (17)	0.0306 (15)	0.0394 (16)	0.0071 (13)	0.0079 (13)	0.0042 (13)
C17	0.058 (2)	0.0395 (17)	0.0384 (17)	0.0193 (15)	0.0086 (15)	0.0000 (14)
C18	0.060 (2)	0.0445 (18)	0.0445 (19)	0.0187 (16)	0.0029 (16)	0.0093 (15)
C19	0.062 (2)	0.0371 (17)	0.055 (2)	0.0244 (16)	0.0060 (17)	0.0118 (15)
C20	0.052 (2)	0.0307 (16)	0.0510 (19)	0.0140 (14)	0.0117 (16)	0.0022 (14)
C21	0.113 (4)	0.073 (3)	0.125 (4)	0.007 (3)	−0.021 (3)	−0.008 (3)
C22	0.093 (3)	0.116 (4)	0.055 (3)	0.039 (3)	0.016 (2)	0.007 (3)

### Geometric parameters (Å, °)

**Table:**

Cu1—O3	1.890 (2)	C7—C8	1.412 (4)
Cu1—O1	1.9038 (19)	C7—H7	0.9300
Cu1—N1	1.933 (2)	C8—C13	1.425 (4)
Cu1—N2	1.941 (2)	C8—C9	1.430 (4)
N1—C7	1.303 (4)	C9—C10	1.398 (4)
N1—C1	1.421 (4)	C10—C11	1.382 (4)
N2—C14	1.303 (3)	C10—H10	0.9300
N2—C2	1.422 (4)	C11—C12	1.401 (4)
O1—C9	1.315 (3)	C12—C13	1.352 (4)
O2—C11	1.351 (4)	C12—H12	0.9300
O2—H2	0.8200	C13—H13	0.9300
O3—C16	1.308 (3)	C14—C15	1.420 (4)
O4—C18	1.368 (4)	C14—H14	0.9300
O4—H4	0.8200	C15—C20	1.416 (4)
O5—C21	1.392 (5)	C15—C16	1.426 (4)
O5—H5	0.8200	C16—C17	1.408 (4)
O6—C22	1.369 (6)	C17—C18	1.373 (4)
O6—H6	0.8200	C17—H17	0.9300
C1—C6	1.385 (4)	C18—C19	1.402 (4)
C1—C2	1.405 (4)	C19—C20	1.354 (5)
C2—C3	1.385 (4)	C19—H19	0.9300
C3—C4	1.381 (5)	C20—H20	0.9300
C3—H3	0.9300	C21—H21A	0.9600
C4—C5	1.374 (5)	C21—H21B	0.9600
C4—H4A	0.9300	C21—H21C	0.9600
C5—C6	1.378 (5)	C22—H22A	0.9600
C5—H5A	0.9300	C22—H22B	0.9600
C6—H6A	0.9300	C22—H22C	0.9600
			
C7···C9^i^	3.185 (4)	C8···C14^ii^	3.232 (4)
			
O3—Cu1—O1	86.42 (8)	C11—C10—C9	121.7 (3)
O3—Cu1—N1	178.39 (9)	C11—C10—H10	119.2
O1—Cu1—N1	94.78 (9)	C9—C10—H10	119.2
O3—Cu1—N2	94.77 (9)	O2—C11—C10	122.8 (3)
O1—Cu1—N2	178.65 (9)	O2—C11—C12	116.9 (3)
N1—Cu1—N2	84.02 (9)	C10—C11—C12	120.3 (3)
C7—N1—C1	122.4 (2)	C13—C12—C11	119.2 (3)
C7—N1—Cu1	124.2 (2)	C13—C12—H12	120.4
C1—N1—Cu1	113.23 (18)	C11—C12—H12	120.4
C14—N2—C2	122.5 (2)	C12—C13—C8	122.7 (3)
C14—N2—Cu1	124.2 (2)	C12—C13—H13	118.7
C2—N2—Cu1	113.32 (18)	C8—C13—H13	118.7
C9—O1—Cu1	127.17 (18)	N2—C14—C15	126.1 (3)
C11—O2—H2	109.5	N2—C14—H14	117.0
C16—O3—Cu1	127.11 (18)	C15—C14—H14	117.0
C18—O4—H4	109.5	C20—C15—C14	118.0 (3)
C21—O5—H5	109.5	C20—C15—C16	118.1 (3)
C22—O6—H6	109.5	C14—C15—C16	123.7 (3)
C6—C1—C2	119.5 (3)	O3—C16—C17	118.3 (3)
C6—C1—N1	125.5 (3)	O3—C16—C15	123.7 (3)
C2—C1—N1	115.1 (2)	C17—C16—C15	118.1 (3)
C3—C2—C1	119.2 (3)	C18—C17—C16	121.4 (3)
C3—C2—N2	126.4 (3)	C18—C17—H17	119.3
C1—C2—N2	114.4 (2)	C16—C17—H17	119.3
C4—C3—C2	120.7 (3)	O4—C18—C17	122.2 (3)
C4—C3—H3	119.7	O4—C18—C19	117.1 (3)
C2—C3—H3	119.7	C17—C18—C19	120.7 (3)
C5—C4—C3	119.8 (3)	C20—C19—C18	118.8 (3)
C5—C4—H4A	120.1	C20—C19—H19	120.6
C3—C4—H4A	120.1	C18—C19—H19	120.6
C4—C5—C6	120.5 (3)	C19—C20—C15	122.8 (3)
C4—C5—H5A	119.7	C19—C20—H20	118.6
C6—C5—H5A	119.7	C15—C20—H20	118.6
C5—C6—C1	120.3 (3)	O5—C21—H21A	109.5
C5—C6—H6A	119.9	O5—C21—H21B	109.5
C1—C6—H6A	119.9	H21A—C21—H21B	109.5
N1—C7—C8	126.3 (3)	O5—C21—H21C	109.5
N1—C7—H7	116.9	H21A—C21—H21C	109.5
C8—C7—H7	116.9	H21B—C21—H21C	109.5
C7—C8—C13	118.0 (3)	O6—C22—H22A	109.5
C7—C8—C9	124.3 (3)	O6—C22—H22B	109.5
C13—C8—C9	117.7 (3)	H22A—C22—H22B	109.5
O1—C9—C10	118.7 (3)	O6—C22—H22C	109.5
O1—C9—C8	123.0 (3)	H22A—C22—H22C	109.5
C10—C9—C8	118.4 (2)	H22B—C22—H22C	109.5
			
O3—Cu1—N1—C7	−144 (3)	Cu1—N1—C7—C8	6.1 (4)
O1—Cu1—N1—C7	−5.2 (2)	N1—C7—C8—C13	177.0 (3)
N2—Cu1—N1—C7	175.4 (2)	N1—C7—C8—C9	−0.8 (4)
O3—Cu1—N1—C1	42 (3)	Cu1—O1—C9—C10	−176.41 (18)
O1—Cu1—N1—C1	−179.98 (18)	Cu1—O1—C9—C8	4.1 (4)
N2—Cu1—N1—C1	0.65 (18)	C7—C8—C9—O1	−4.7 (4)
O3—Cu1—N2—C14	−1.3 (2)	C13—C8—C9—O1	177.5 (2)
O1—Cu1—N2—C14	150 (4)	C7—C8—C9—C10	175.8 (3)
N1—Cu1—N2—C14	177.7 (2)	C13—C8—C9—C10	−2.0 (4)
O3—Cu1—N2—C2	179.91 (18)	O1—C9—C10—C11	−178.6 (2)
O1—Cu1—N2—C2	−28 (4)	C8—C9—C10—C11	0.9 (4)
N1—Cu1—N2—C2	−1.15 (18)	C9—C10—C11—O2	−179.7 (3)
O3—Cu1—O1—C9	179.2 (2)	C9—C10—C11—C12	0.7 (4)
N1—Cu1—O1—C9	0.3 (2)	O2—C11—C12—C13	179.3 (3)
N2—Cu1—O1—C9	27 (4)	C10—C11—C12—C13	−1.0 (4)
O1—Cu1—O3—C16	−172.9 (2)	C11—C12—C13—C8	−0.2 (5)
N1—Cu1—O3—C16	−34 (3)	C7—C8—C13—C12	−176.3 (3)
N2—Cu1—O3—C16	6.5 (2)	C9—C8—C13—C12	1.7 (4)
C7—N1—C1—C6	5.7 (5)	C2—N2—C14—C15	177.7 (3)
Cu1—N1—C1—C6	−179.4 (3)	Cu1—N2—C14—C15	−1.1 (4)
C7—N1—C1—C2	−174.9 (2)	N2—C14—C15—C20	−176.1 (3)
Cu1—N1—C1—C2	0.0 (3)	N2—C14—C15—C16	−0.5 (5)
C6—C1—C2—C3	−0.1 (4)	Cu1—O3—C16—C17	170.0 (2)
N1—C1—C2—C3	−179.5 (2)	Cu1—O3—C16—C15	−9.4 (4)
C6—C1—C2—N2	178.5 (3)	C20—C15—C16—O3	−178.4 (3)
N1—C1—C2—N2	−0.9 (4)	C14—C15—C16—O3	6.0 (5)
C14—N2—C2—C3	1.0 (4)	C20—C15—C16—C17	2.1 (4)
Cu1—N2—C2—C3	179.9 (2)	C14—C15—C16—C17	−173.5 (3)
C14—N2—C2—C1	−177.4 (2)	O3—C16—C17—C18	179.2 (3)
Cu1—N2—C2—C1	1.4 (3)	C15—C16—C17—C18	−1.3 (5)
C1—C2—C3—C4	−0.7 (5)	C16—C17—C18—O4	178.6 (3)
N2—C2—C3—C4	−179.0 (3)	C16—C17—C18—C19	−0.3 (5)
C2—C3—C4—C5	1.1 (5)	O4—C18—C19—C20	−177.8 (3)
C3—C4—C5—C6	−0.9 (6)	C17—C18—C19—C20	1.1 (5)
C4—C5—C6—C1	0.1 (6)	C18—C19—C20—C15	−0.2 (5)
C2—C1—C6—C5	0.3 (5)	C14—C15—C20—C19	174.4 (3)
N1—C1—C6—C5	179.7 (3)	C16—C15—C20—C19	−1.4 (5)
C1—N1—C7—C8	−179.6 (2)		

Symmetry codes: (i) −*x*+1, −*y*+2, −*z*; (ii) −*x*+2, −*y*+2, −*z*.

### Hydrogen-bond geometry (Å, °)

**Table:**

*D*—H···*A*	*D*—H	H···*A*	*D*···*A*	*D*—H···*A*
O2—H2···O5^iii^	0.82	1.91	2.704 (3)	163
O4—H4···O6^iv^	0.82	1.82	2.602 (4)	159
O5—H5···O4^v^	0.82	1.97	2.788 (3)	176
O6—H6···O1	0.82	2.18	2.777 (3)	130
O6—H6···O3	0.82	2.34	3.037 (4)	144

Symmetry codes: (iii) *x*, *y*+1, *z*; (iv) −*x*+2, −*y*+2, −*z*+1; (v) −*x*+2, −*y*+1, −*z*+1.
